# Analysis of Molecular Disordering Processes in the Phase Transition of Liquid Crystals Observed by Patterned-Illumination Time-Resolved Phase Microscopy

**DOI:** 10.3390/ma14195491

**Published:** 2021-09-23

**Authors:** Nozomi Sato, Kenji Katayama

**Affiliations:** Department of Applied Chemistry, Chuo University, Kasuga, Bunkyo-ku, Tokyo 112-8551, Japan; a16.357r@g.chuo-u.ac.jp

**Keywords:** liquid crystals, time-resolved imaging, phase transition, dye-doped liquid crystals

## Abstract

The initial processes of the phase transition dynamics of liquid crystals (LCs) subject to UV pulse irradiation were clarified using a nanosecond time-resolved imaging technique called pattern-illumination time-resolved phase microscopy (PI-PM). Two types of LCs were studied: a photo-responsive LC and dye-doped LCs. We found two steps of molecular disordering processes in the phase transition, namely local disordering proceeding anisotropically, followed by the spreading of the isotropic phase. These two processes were separated for a photo-responsive LC while being simultaneously observed for the dye-doped LCs. It was found that the photomechanical dyes induced the phase transition process faster than the photothermal dyes.

## 1. Introduction

In LCs, there is long-range ordering of the molecules, orienting all the molecules approximately in the same direction, called the director, while the molecules can flow as in a standard liquid. This anisotropic structure provides varieties of unique thermal, optical, electric, and mechanical properties. Liquid crystals (LCs) have been used for display purposes in industry, and the LC state can be found in living matter [1]. A phase transition, transforming the LC into the isotropic phase, can be induced by increasing the temperature. However, the application of an electric or magnetic field can be used to reorient the director. The onset of this process is characterized by the Freedericksz threshold. On the other hand, it was first reported by Haas et al. that the phase could be controlled in photo-responsive LCs by light irradiation [2]. This research was extended for the control of the phase and alignment by adding a small amount of photo-responsive guest molecules. Ichimura et al. demonstrated the control of LC alignment by the structural change of the azobenzene moiety attached to the substrate [3]. Ikeda et al. proposed the phase control of the LCs at a lower temperature than the phase transition temperature [4,5,6,7].

The interaction between the guest molecules and the host LCs has attracted much attention; the photomechanical motion of molecules could move the macroscopic structure pioneered by Ikeda and Yu [8]. Motion caused by the photomechanical force was demonstrated for wavy motion [9], flow was controlled by a photomechanical tube [10], and molecular manipulation by the control of order parameter via polymerization [11,12]. In addition, a huge enhancement of the optical nonlinearity was found and its mechanism and applications were extensively studied [13,14,15,16,17,18,19,20,21,22,23,24,25,26,27].

From the mechanistic viewpoint on the phase transition, the molecular dynamics during the process must be clarified, and time-resolved measurements must be used for the LC dynamics. Khoo et al. have led the field of study by using grating-type photoexcitation and clarifying the temperature and flow-induced orientation [28], and the phase transition dynamics were studied using similar experiments [29,30,31]. However, the photo-induced orientation change or the phase transition is highly dependent on the light irradiation conditions for the dye-doped LCs, and was observed with time-resolved imaging [32]. It was difficult to precisely control the optically induced change due to the inhomogeneity of the focused light irradiation due to the random orientation which, in turn, was used for the light source of microscopy [33]. We developed a heterodyne transient grating technique, where the irradiation light was homogeneous in a broad region and could provide a reproducible response, and the multiple processes depending on the light intensity were clarified [34]. The phase transition and photo-induced orientation change were distinguished and the anisotropic phase transition/recovery was clarified by the observation of the extraordinary and ordinary refractive index change [35], and also revealed that the front surface of phase transition moved at ~100 m/s [36]. In recent years, we have studied the origin of the optical nonlinearity from the dynamics of the LCs by using time-resolved methods and found that the enhancement occurred on the millisecond time scale [37]. Furthermore, a new time-resolved imaging technique was developed, and the origin was clarified by visualizing the images of the optically induced flow of the LCs [38].

In this study, a time-resolved imaging technique called the PI-PM method [39], with a time resolution of a few nanoseconds, was applied for the study of the phase transition dynamics of the photochemical phase transition of LCs. This method could directly visualize the phase transition process. We clarified the initial dynamics of the collective behavior of the LC molecules leading to the phase transition and found that the phase transition proceeded via single or multiple steps, and the process depended on the director direction. We could successfully explain the processes by a model function representing the autocatalytic reaction.

## 2. Theory and Method

The principle of the PI-PM method was described in previous papers and is shown in Figure S1 in Supporting Information (SI) [38,39]. In brief, an arbitrary pump light pattern is irradiated to a sample, causing photoisomerization and the following molecular rotation and disordering for the LCs. Because these processes induce a refractive index change, a refractive-index pattern was formed as the same pattern of the pump beam. The refractive index was imaged by the self-imaging technique [40], and a temporal change in the refractive index images was recorded.

In the case of LCs, the physical origin of the photo-induced refractive index change is typically divided into three components:

(1) $$∆n\left(t\right)=∆n_{T}\left(t\right)+∆n_{S}\left(t\right)$$

where the terms on the right side correspond to the index changes due to temperature increase and order parameter. In previous studies of the photo-induced dynamics of LCs, the $∆n_{T}\left(t\right)$ term was caused by the density change due to the disordering [41], while the $∆n_{S}\left(t\right)$ term was the ordering change of the LCs. The main origin of the disordering dynamics of the LC is the $∆n_{S}\left(t\right)$ term, whose sign is dependent on whether the polarization of the probe light is perpendicular or parallel to the LC director axis, while the sign of the $∆n_{T}\left(t\right)$ term is not dependent on the polarization of the probe light [34].

Since the orientational change of molecular assembly propagates in a collective manner owing to the long-range molecular interaction, the photo-induced disordered region acts as a catalyst for the following disordering in the phase transition. This process is similar to the autocatalytic reaction in chemistry, where the reactant itself works as a catalyst [42,43]. A time response for the autocatalytic reaction can be described by a sigmoidal curve and can be fitted with a logistic function [44,45,46]. Assuming that the change in the refractive index is proportional to the disordered area of the phase transition region, the temporal change in the refractive index can be fitted with a logistic function [47]. The area of the disordered region, y, is represented as [48]

(2) $$y\left(t\right)=\frac{y_{\max}-y_{\min}}{1+e^{-kt}}+y_{\min}$$

where k, $y_{\min}$, and $y_{\max}$ are the rate constant, the phase transition region at time 0, and the saturated phase transition region, respectively. In the analysis, instead of using Equation (2), we used Equation (3) because our experimental data were extended into a logarithmic time scale.

(3) $$y=\frac{y_{\max}-y_{\min}}{1+e^{-A\left(\log t-\log t_{\mathrm{half}}\right)}}+y_{\min}$$

where $\log t_{\mathrm{half}}$ corresponds to the time when the signal intensity becomes equal to half of $y_{\max}-y_{\min}$. In this analysis, A is an adjusting parameter for the fit, and the physical meaning of the phase transition time is included in $\log t_{\mathrm{half}}$.

**Figure 1:**
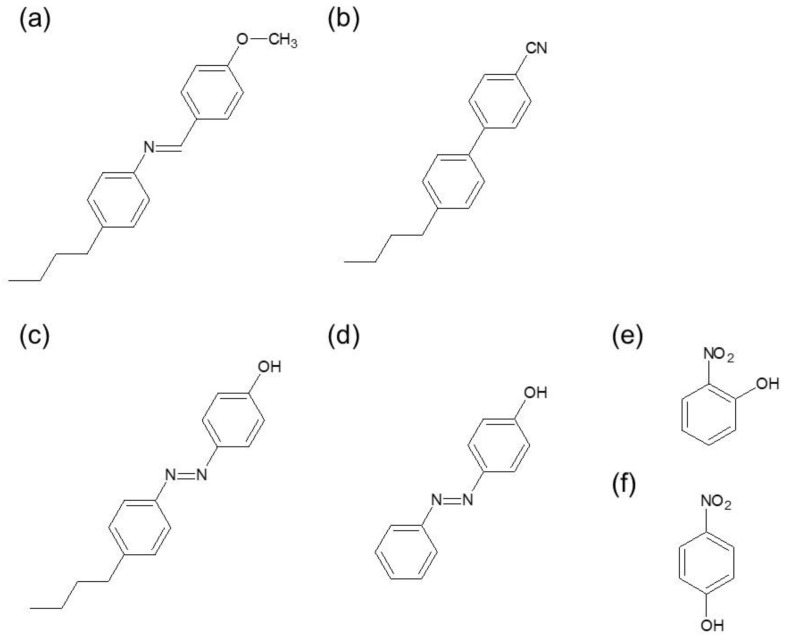
The molecular structures of the LCs and photo-responsive guest molecules are shown: (**a**) 4-methoxybenzylidene-4-n-butylaniline (MBBA), (**b**) 4-cyano-4-pentylbiphenyl (5CB), (**c**) 4-hydroxyazobenzene (PPAP), (**d**) 4-butyl-4′-hydroxyazobenzene (BHAB), (**e**) 2-nitrophenol (o-NP), and (**f**) 4-nitrophenol (p-NP).

We used 4-methoxybenzylidene-4-n-butylaniline (MBBA) as photo-responsive nematic LCs (Figure 1a). MBBA has a C-N double bond, which causes trans–cis photoisomerization by UV irradiation. The formation of cis isomers scatters the alignment of molecules, causing disordering. Concerning the study of phase transition dynamics for the dye-doped LC, 4-cyano-4-pentylbiphenyl (5CB) was used as a host nematic LC (Figure 1b). The photo-responsive guest molecules used were 4-hydroxyazobenzene (PPAP), 4-butyl-4′-hydroxyazobenzene (BHAB), 2-nitrophenol (o-NP), and 4-nitrophenol (p-NP) (Figure 1c–f). With regard to the azo-doped LCs (photomechanical system), not only was there a photothermal effect, but the photomechanical effect owing to the trans–cis photoisomerization of guest molecules played a role in the host LCs disordering. On the other hand, o-NP and p-NP induced only photothermal effects, leading to disordering (photothermal system).

The optical configuration of the PI-PM is shown in Figure S1. A stripe pattern of UV light with the grating spacing of 60 μm was irradiated as a pump light, and by matching the probe polarization and the LC director, the images of the change in the extraordinary refractive index ($∆n_{e}\left(t\right)$) were obtained. Those in the ordinary refractive index ($∆n_{o}\left(t\right)$) were measured by setting them perpendicularly. The pump polarization was set parallel to the LC director. The irradiation intensity of the pump light was changed in the range of 2.67–6.58 mJ/pulse, and the probe light intensity was 0.02 mJ/pulse.

MBBA, 5CB, PPAP, BHAB, p-NP (Tokyo Kasei, Tokyo, Japan), and o-NP (FUJIFILM Wako Pure Chemical, Osaka, Japan) were used as purchased without further purification. The concentrations of the guest dyes were adjusted so that the absorbance was 0.1~0.2 at the pump wavelength for the polarization parallel to the LC director to ensure light absorption throughout the depth direction. The sample was put into an LC cell (E.H.C, Tokyo, Japan) with a sample thickness of ~3 μm, an ITO layer, and a rubbed polyimide film for a planar alignment inside. The LC cell was covered by an aluminum frame, and the temperature was controlled by a heater controller (TC200, Thorlabs, USA). The temperature was set at 30.0–31.0 °C. The phase transition temperature of 5CB from the nematic to the isotropic state was 35.1 °C. The ITO and polyimide layer in the cell had a minor absorption at the pump wavelength, but the effect was negligible compared with the absorption of MBBA and dyes, which was confirmed because no refractive index change was observed for pure 5CB under the same experimental condition [49].

## 3. Results

### 3.1. Photo-Responsive LC

**Figure 2:**
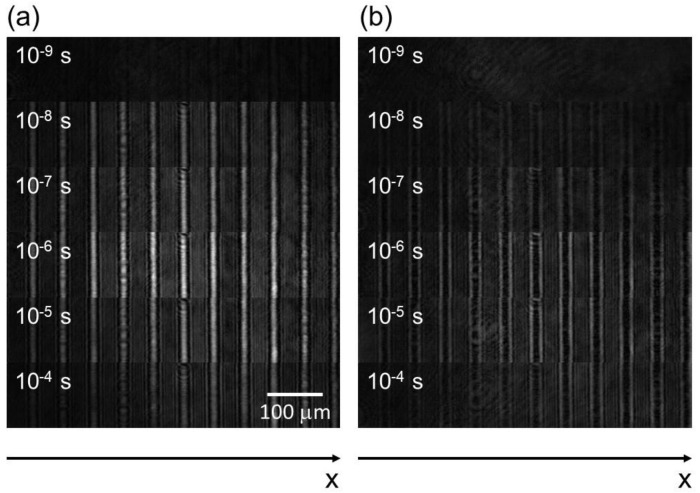
The image sequences of MBBA recorded by the PI-PM method are shown: (**a**) $∆n_{e}\left(t\right)$ and (**b**) $∆n_{o}\left(t\right)$ images were captured when the probe polarization was parallel or perpendicular to the director axis, respectively. The pump intensity was 4.22 mJ/pulse.

Figure 2 shows the image sequences of the $∆n_{e}\left(t\right)$ and $∆n_{o}\left(t\right)$ responses for MBBA at a pump intensity of 4.22 mJ/cm^2^. In both of the image sequences, the contrast of the stripe pattern of the refractive index change was observed. The contrast became larger and took maxima around 10^−6^ s, and disappeared with a decay of about 10^−4^ s. The white region in Figure 2a corresponds to a decrease of the refractive index, while the black region in Figure 2b indicates an increase. The opposite sign of the refractive index change was reasonable, considering the sign of the refractive index change was due to the order parameter variation.

**Figure 3:**
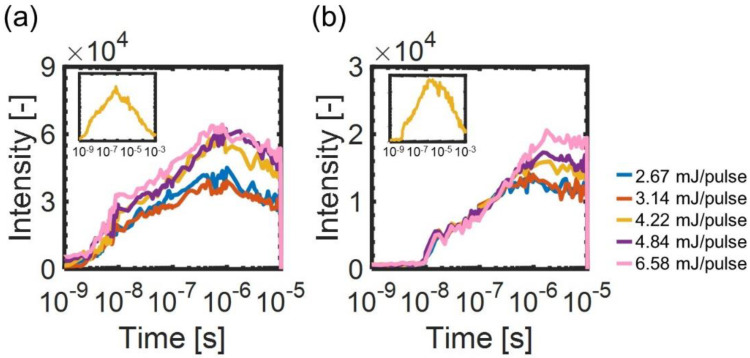
The temporal changes of the stripe amplitudes of the refractive index images for different intensities of the pump light: (**a**) $∆n_{e}\left(t\right)$ (**b**) $∆n_{o}\left(t\right)$ responses. The pump light intensities were 2.67, 3.14, 4.22, 4.84, and 6.58 mJ/pulse. The insets show temporal changes until a millisecond at a pump intensity of 4.22 mJ/cm^2^. The time axis is shown in a logarithmic timescale.

We recorded $∆n_{e}\left(t\right)$ and $∆n_{o}\left(t\right)$ images on the different pump light intensities. To assign the components in the responses, the stripe amplitude was obtained by applying a Fourier transform in the x-direction of the refractive index images. The temporal changes of the amplitude are shown in Figure 3 and correspond to the temporal changes of the refractive index change [50]. Insets in Figure 3 show the responses until a millisecond for the pump intensity of 4.22 mJ/pulse.

From the overall responses shown in the insets of Figure 3, the signal intensity for each probe polarization increased until several microseconds, followed by a decay until a millisecond. The rising component corresponds to the disordering and the subsequent decay component corresponds to the ordering processes, with the result agreeing well with the previous results [35,36]. At any pump intensity, $∆n_{e}\left(t\right)$ responses started to increase within the time resolution (3 ns). On the other hand, $∆n_{o}\left(t\right)$ responses started to rise after ~10 ns. Furthermore, we found that the responses for each probe polarization consisted of two rising components: the first component on the order of 10^−8^ s, followed by the second component on the order of 10^−6^ s.

With regard to the phase transition of LCs, a partial phase transition was proposed in previous research [51]. We also posited a similar proposal: a local phase transition was induced nearby the photo-excited molecules, followed by a phase transition in an entire region. Based on the above results and previous research, we suppose that the two-step phase transition process occurred: a local phase transition process corresponding to anisotropic disordering in the LC orientational direction (~10^−8^ s), and the subsequent phase transition process induced by an isotropic expansion of the disordered region (~10^−6^ s). In the first disordering process, the phase transition was induced in the director direction, followed by that in the perpendicular direction. These findings and results of this study indicate that the molecular interaction in the director direction was first lost, and then the disordering followed in the other directions.

**Figure 4:**
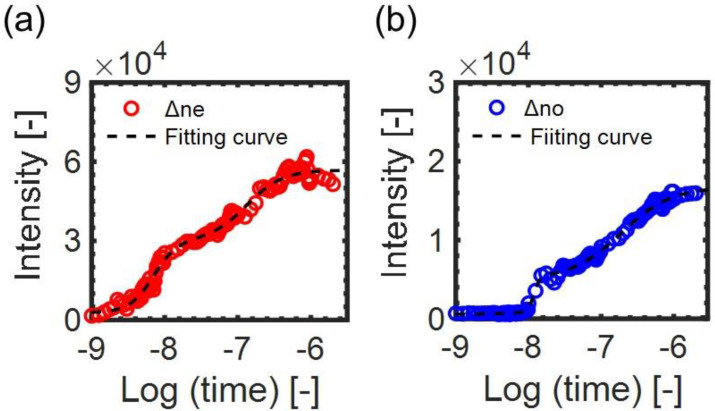
The examples of the fitting curves for (**a**) $∆n_{e}\left(t\right)$ and (**b**) $∆n_{o}\left(t\right)$ responses by a model function of Equation (4): the response curves for the pump intensity 4.22 mJ/pulse are shown. The fitting parameters were (**a**) $y_{0}=3.81\times 10^{3}$, $y_{1}=3.02\times 10^{4}$, $y_{2}=2.66\times 10^{4}$, $\log t_{\mathrm{half},1}=-8.15$, $\log t_{\mathrm{half},2}=-6.88$ and (**b**) $y_{0}=6.32\times 10^{2}$, $y_{1}=5.62\times 10^{3}$, $y_{2}=9.90\times 10^{3}$, $\log t_{\mathrm{half},1}=-7.92$, $\log t_{\mathrm{half},2}=-6.78$.

Since each step of the phase transition process is regarded as one of the auto-catalytic reactions, we extended a logistic function model of Equation (3) to a function for a two-step auto-catalytic reaction. The model function is shown as

(4) $$y=\left(\frac{y_{1}-y_{0}}{1+e^{-A_{1}\left(\log t-\log t_{\mathrm{half},1}\right)}}\right)+\left(\frac{y_{2}}{1+e^{-A_{2}\left(\log t-\log t_{\mathrm{half},\;2}\right)}}\right)+y_{0}$$

where $y_{1}$, $y_{2}$, and $y_{0}$ are the areas of the disordering regions caused by each disordering process, $\log t_{\mathrm{half},\;1}$ and $\log t_{\mathrm{half},\;2}$ are the log half-times for each disordering process, and $A_{1}$ and $A_{2}$ are the adjusting parameters. Figure 4 shows examples of the fitting curves for the responses with Equation (4). We used the data points until the signal response was saturated, and several extra data points were added so that the fitting result was not affected by the noise fluctuation. Reasonable fitting curves of $∆n_{e}\left(t\right)$ and $∆n_{o}\left(t\right)$ responses were obtained. In both cases, the coefficients of determination $R^{2}>0.98$.

**Figure 5:**
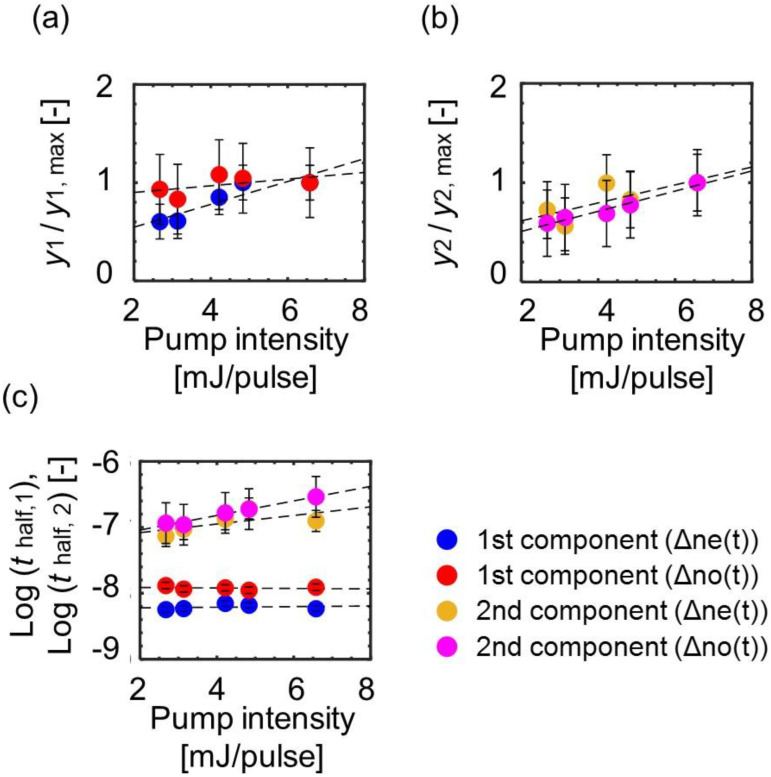
The parameter dependences on the probe polarization and the pump intensity: the area of the disordered region when (**a**) the first and (**b**) the second disordering processes finished, whose parameter values were divided by those at a pump intensity of 6.58 mJ/cm^2^; (**c**) the log half-time for each disordering process.

To understand the probe polarization and pump intensity dependences, the responses were fitted with the model function. Figure 5 shows the fitting parameters’ dependences on the polarization and pump intensity, where $y_{1}$ and $y_{2}$ were normalized by the values at the pump intensity 6.58 mJ/cm^2^ ($y_{1,\;\max}$ and $y_{2,\;\max}$) to confirm the dependence on the polarization. Figure 5a,b demonstrate that y_1_ and y_2_ depended on the pump intensity, except for the first component of $∆n_{o}$. Since they represent the area of the disordered region when each process has finished, the result means that the area of the disordered region increased anisotropically as the pump intensity increased in the first process, while it increased isotropically in the second process. Figure 5c demonstrates that the log half-times did not depend on pump intensity for the first process, while the second process became slower as the pump intensity increased. This result indicates that the initial processes proceeded around the same time for different pump intensities. On the other hand, the second phase transition took more time until the end of the disordering process because the region of the phase transition expanded in a larger area as pump intensity increased. The adjusting parameters remained to be similar values for different pump intensities (Figure S2).

**Figure 6:**
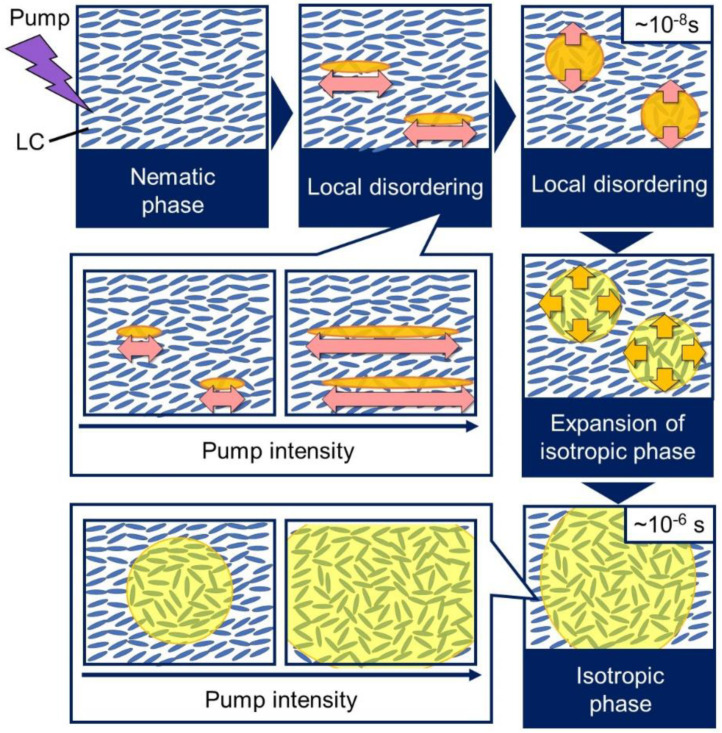
The schematic illustration of the photo-induced phase transition model is summarized.

Figure 6 shows a model drawing of the phase transition dynamics based on the above results. There are two types of phase transition processes: local disordering occurred first, followed by the second phase transition. In the local disordering process, the disordering extended into the director axis as the pump intensity increased, while the disordering in the perpendicular direction to the director remained the same. The second process corresponds to the expansion of the isotropic region; the area and time for the phase transition increased as the pump intensity increased because it took time for the larger area of the phase transition and because the region expansion speed does not depend on the pump intensity [36].

### 3.2. Dye-Doped LC

**Figure 7:**
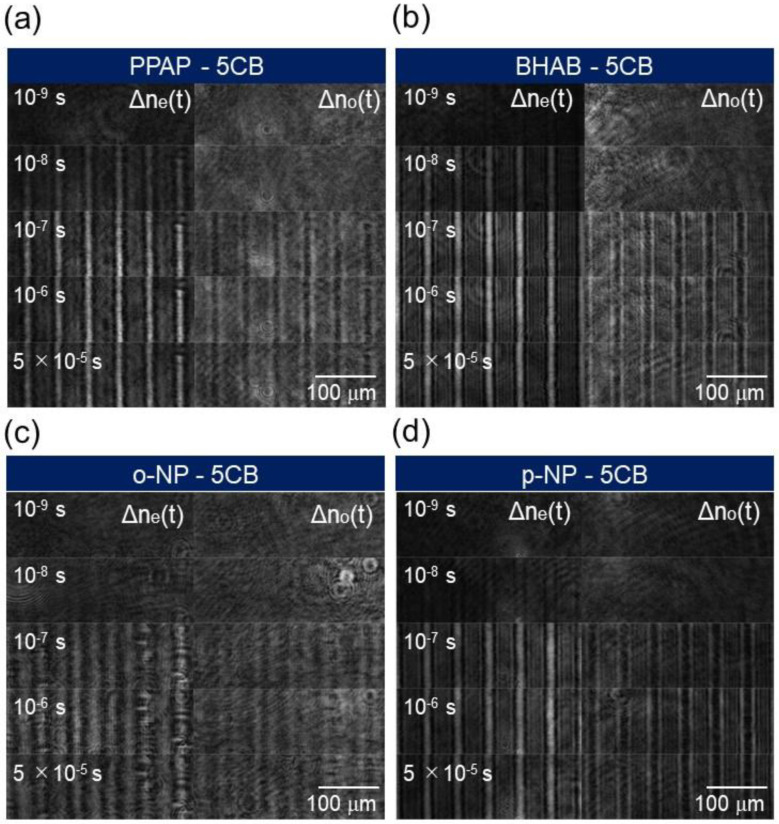
The image sequences corresponding to $∆n_{e}\left(t\right)$ and $∆n_{o}\left(t\right)$ of each sample recorded by the PI-PM method are shown. The pump intensity was 2.67 mJ/pulse. The samples were 5CB doped with (**a**) PPAP, (**b**) BHAB, (**c**) o−NP, and (**d**) p−NP. The examples of overall image sequences are shown in Figure S3a.

In this section, the phase transition dynamics for the dye-doped LCs are shown. Figure 7 shows the image sequences of the $∆n_{e}\left(t\right)$ and $∆n_{o}\left(t\right)$ responses for 5CB doped with different dyes at a pump intensity of 2.67 mJ/cm^2^. Similar to the results for MBBA in Figure 3, the contrast became larger until ~1 μs, followed by a gradual decay (~100 μs). The image sequence and the corresponding time response of the refractive index change for the BHAB-doped 5CB for the longer time region is shown in Figure S3 in SI. For all the dye molecules, the sign of the responses between the $∆n_{e}\left(t\right)$ and $∆n_{o}\left(t\right)$ was opposite, reflecting that the refractive index change due to 5CB was observed.

**Figure 8:**
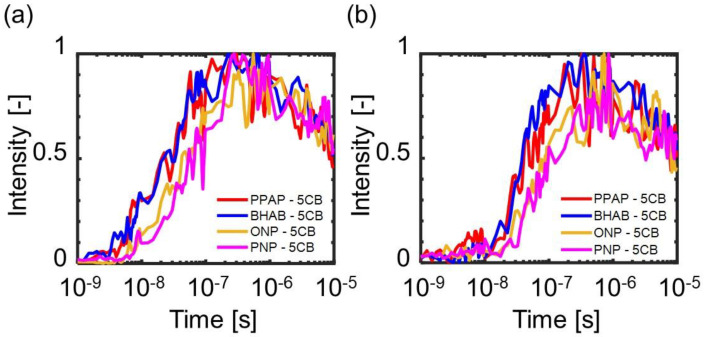
The temporal changes of the amplitudes of the stripe contrast in the refractive index images for each sample: (**a**) $∆n_{e}\left(t\right)$ response; (**b**) $∆n_{o}\left(t\right)$ response. The time axis is shown in a logarithmic time scale. The pump intensity was 2.67 mJ/pulse. The samples were 5CB doped with PPAP, BHAB, o−NP, and p−NP. The examples of overall responses are shown in Figure S3b.

Figure 8 shows the temporal responses of the amplitudes for the refractive index images for each sample. $∆n_{e}\left(t\right)$ responses showed a rise first, followed by the rise of $∆n_{o}\left(t\right)$ responses for all the samples. This result is similar to the result of the MBBA as shown in Figure 3. The responses for each probe polarization showed a single rising component until ~1 μs, while the response for MBBA showed a two-step rise (Figure 3). This result indicates that the local disordering and the expansion of the phase transition were not separated, and both processes were mixed in the case of the dye-doped LCs. Similar to the MBBA case, the phase transition was induced first in the director direction, followed by that in the perpendicular direction.

**Figure 9:**
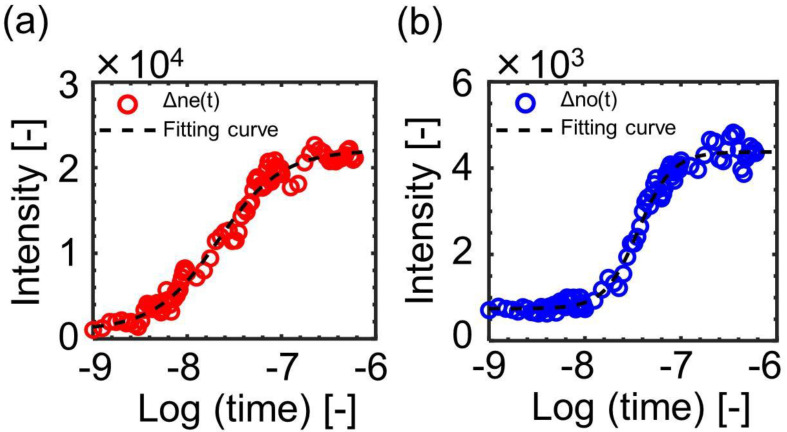
The examples of the fitting curves for the (**a**) $∆n_{e}\left(t\right)$ and (**b**)$\;∆n_{o}\left(t\right)$ responses by a model function of Equation (3). The data correspond to the responses for 5CB doped with BHAB. The pump intensity was 2.67 mJ/pulse. The fitting parameters were (**a**) $y_{max}=2.09\times 10^{4}$, $y_{min}=1.38\times 10^{3}$, $\log t_{\mathrm{half}}=-7.71$, (**b**) $y_{max}=4.23\times 10^{3}$, $y_{min}=7.62\times 10^{2}$, $\log t_{\mathrm{half}}=-7.46$.

Thus, we assumed that an overall phase transition process corresponded to a single auto-catalytic reaction and fitted the responses with a single logistic model function (Equation (3)). Figure 9 shows examples of the fitting curves for the responses. The reasonable fitting curves of the $∆n_{e}\left(t\right)$ and $∆n_{o}\left(t\right)$ responses were obtained with $R^{2}>0.94$.

**Figure 10:**
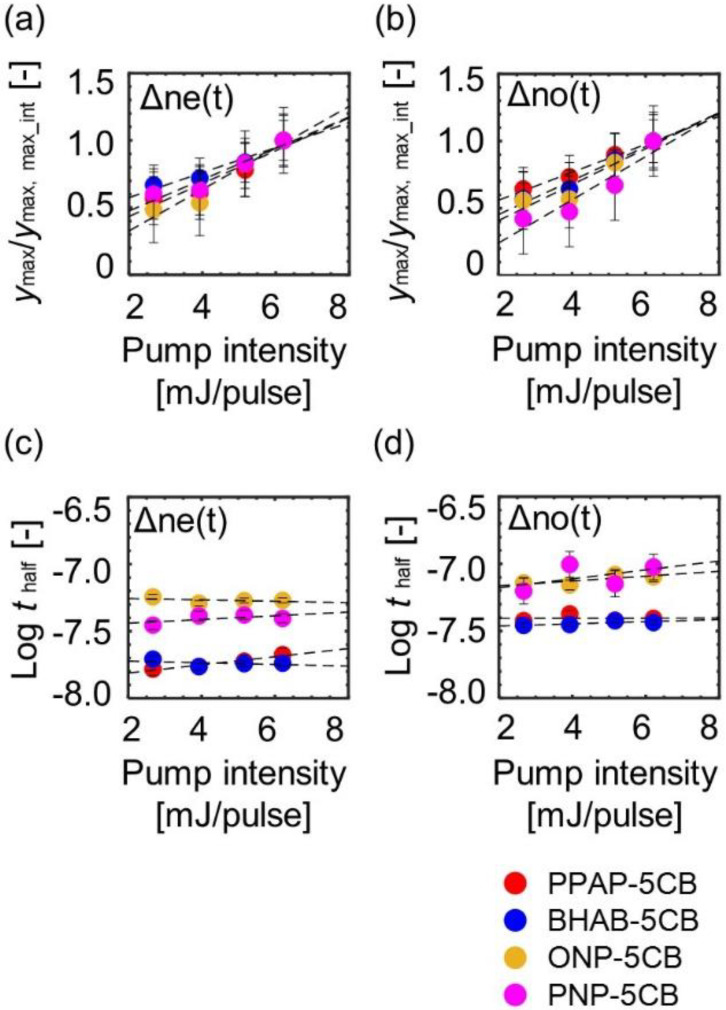
The parameter dependences on the probe polarization and the pump intensity: (**a**,**b**) the area parameters of the disordered region when the disordering processes finished, whose parameter values were divided by a pump intensity of 6.21 mJ/cm^2^; (**c**,**d**) the log half-time for the disordering process. The pump light intensities were 2.67, 3.93, 5.16, and 6.21 mJ/pulse.

Figure 10 shows the fitting parameters’ dependences on the polarization and pump intensity, where $y_{\max}$ were normalized by the values at the pump intensity, 6.21 mJ/cm^2^ ($y_{\max,\;\max\_\mathrm{int}}$). From Figure 10a,b, these parameters depended on the pump intensity, indicating that the area of the disordered region increased isotropically as pump intensity increased. Figure 10c,d show that the log half-time for the photomechanical dyes was faster than that for the photothermal dyes in any pump intensity, meaning that the photomechanical molecules induced faster disordering of LC than the photothermal molecules (the information of the adjusting parameters is included in the supporting information (Figure S4)).

**Figure 11:**
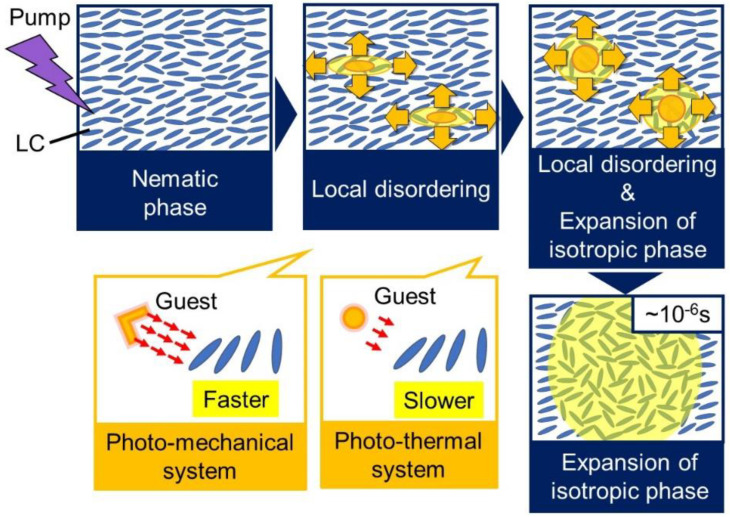
The schematic model illustration of the phase transition for the dye-doped LCs is summarized.

We proposed a model of the phase transition dynamics for the dye-doped LC based on the above results (Figure 11). The phase transition occurred anisotropically and was the same process as that observed for MBBA (top row in Figure 6); however, the local disordering and the following expansion of the isotropic phase were not separated in this case, although these two processes were separated for MBBA (Figure 6). Since the guest molecules in the photomechanical system induced not only the photothermal, but also the photochemical effect in the phase transition owing to photoisomerization, the origin of the faster phase transition in the photomechanical system is assumed due to the change in the molecular structure of the guest molecules by the pump light irradiation.

## 4. Conclusions

We studied the initial processes of the phase transition dynamics of photo-responsive and dye-doped LCs by PI-PM, where the image sequences of the refractive index change were observed with a high time resolution. Two steps of molecular disordering processes leading to the phase transition were observed: local disordering extending anisotropically, followed by the expansion of the isotropic phase. These two processes were separated for a photo-responsive LC; the first step until 10^−8^ s and the second one until 10^−6^ s. They proceeded at the same time and were observed until 10^−6^ s for the dye-doped LCs. The processes were explained by the model function representing the autocatalytic reaction. It was clarified that the photomechanical dyes could induce the phase transition process faster than photothermal ones due to the photoisomerization. These insights into understanding the processes will help in improving the phase control of LCs and LC devices.

## Data Availability

All the data are available on request.

## Supplementary Materials

The following are available online at https://www.mdpi.com/article/10.3390/ma14195491/s1, Figure S1: Experimental setup, Figure S2: Adjusting parameters for MBBA, Figure S3: Overall image sequences and $∆n_{e}\left(t\right)$ and $∆n_{o}\left(t\right)$ responses of 5CB doped with BHAB, Figure S4: Adjusting parameters in the response fitting for dye-doped LCs.
