# Supplementary material for: Analysis of Molecular Disordering Processes in the Phase Transition of Liquid Crystals Observed by Patterned-Illumination Time-Resolved Phase Microscopy
Source: Materials (Basel). 2021 Sep 23;14(19):5491. doi: 10.3390/ma14195491 (PMC8509586; doi:10.3390/ma14195491)
Supplement: Supplementary file 1 [file materials-14-05491-s001.zip › materials-1371222-supplementary.pdf]

Supporting Information

Analysis of Molecular Disordering Processes in the Phase Transition of Liquid Crystals Observed by Patterned-illumination Time-resolved Phase Microscopy

Nozomi Sato[1], Kenji Katayama[1*]

1 Department of Applied Chemistry, Chuo University, Tokyo 112-8551, Japan

*Corresponding authors:

K. Katayama, Phone: +81-3-3817-1913, E-mail: kkata@kc.chuo-u.ac.jp

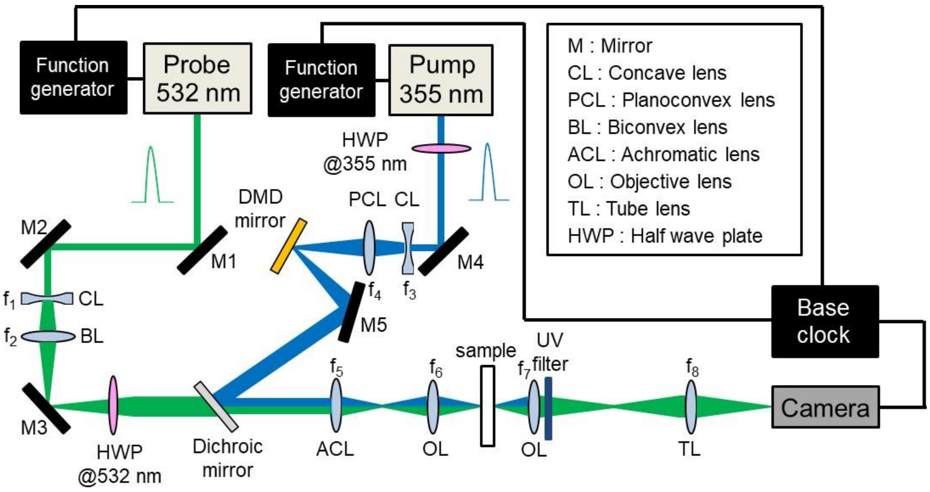

Figure S1. The schematic overview of the optical setup of time-resolved phase microscope is shown. A pump pulse was first reflected at a digital micromirror device (DMD) mirror with an arbitrary pattern. Another probe pulse was irradiated onto the sample. For the imaging purpose, an objective lens and a tube lens were used before the CMOS camera. For the pattern illumination, a DMD (Light Crafter 4500, Texas Instruments) was used. The pump light was reflected at the DMD mirror to change the intensity pattern same as the pattern on a computer. The image of the DMD mirror was relayed with a lens (f = 100 mm) and an objective lens (LUCPLFLN20x, Olympus) to irradiate the same pattern reduced in size (1/14) onto a sample. The pulsed illumination light was collimated with the pump light at the dichroic mirror and illuminated onto a sample. The transmitted light was imaged by an objective lens (LUCPLFLN20x, Olympus) and a tube lens (TTL180-A. Thorlabs). A CMOS camera (MV1-D1024E-160, Photon Focus) had a sensor area of 10.9x10.9mm (1024x1024 pixel), and the central region in the vertical direction (200x1024 pixel) was recorded to reduce the burden of the computer processing. The diameter of the irradiated area by the pump pulse was 0.5 mm. By varying the time delay between the pump and probe pulse, a sequence of images was stored into a computer. The time resolution was limited only by the pulse width of the pump and probe lights, 5 ns. The pump light was the third harmonics of a Nd:YAG pulse laser (pulse width: 5 ns, wavelength: 355 nm) (GAIA, Rayture Systems). The probe light was the second harmonics of an Nd:YAG pulse laser (pulse width: 5 ns, wavelength: 532 nm) (GAIA, Rayture Systems). The timing of these pulses was controlled by two function generators (WF1968, NF) triggered by a base clock (DF1906, NF). Each function generator controlled both the timing of the flash lamp and the Q-switch with a time resolution of 100 ps. The pump polarization and the LC director were matched by a half wave plate (WPH05M-355, Thorlabs). The probe polarization was switched by rotating a half wave plate (WPH05M-532, Thorlabs). $\Delta n_e(t)$ was observed when the probe polarization was parallel to the LC director, and $\Delta n_o(t)$ was measured with the probe polarization perpendicular to it. The pump light intensity was 0.67 mJ/pulse, and the probe light intensity was 0.02 mJ/pulse, respectively.

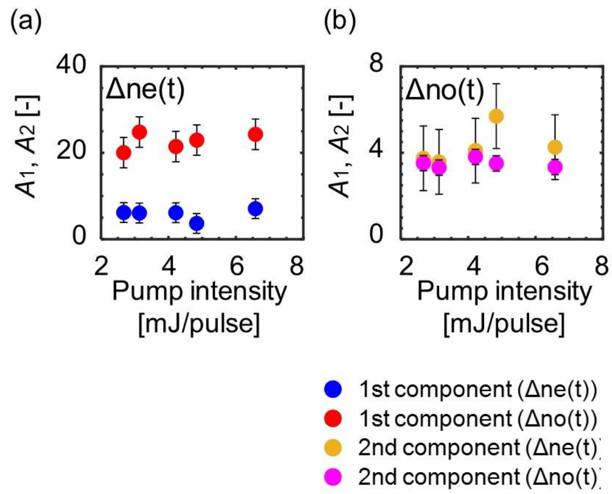

Figure S2. The adjusting parameters for (a) $\Delta n_{\mathrm{e}}(t)$ and (b) $\Delta n_{\mathrm{o}}(t)$ for MBBA are shown. The pump light intensities were 2.67, 3.14, 4.22, 4.84, and 6.58 mJ/pulse.

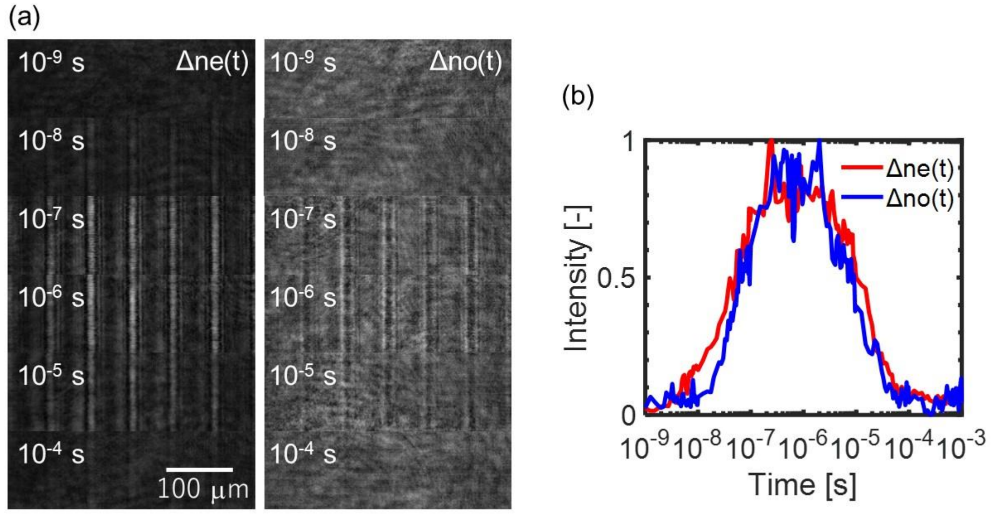

Figure S3. (**a**) The overall image sequences corresponding to $\Delta n_{\mathrm{e}}(t)$ and $\Delta n_{\mathrm{o}}(t)$ of 5CB doped with BHAB are shown. The pump intensity was 2.67 mJ/pulse. (**b**) The temporal changes of the amplitudes of the stripe contrast in the refractive index images are shwon. The time axis is shown in a logarithmic timescale.

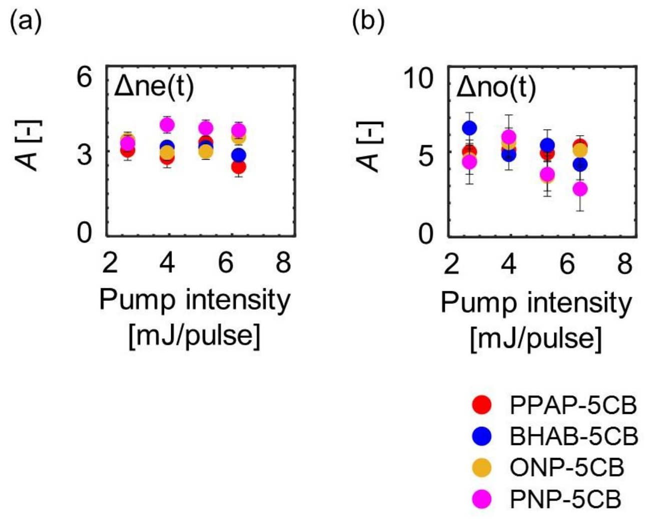

Figure S4. The adjusting parameters for (**a**) $\Delta n_e(t)$ and (**b**) $\Delta n_o(t)$ for the dye-doped LCs are shown. The pump light intensities were 2.67, 3.93, 5.16 and 6.21 mJ/pulse. The samples were 5CB doped with PPAP, BHAB, o-NP, and p-NP.
